# The complete chloroplast genome of *Indigofera stachyodes* (Fabaceae), a traditional Chinese medicinal plant

**DOI:** 10.1080/23802359.2022.2050472

**Published:** 2022-03-09

**Authors:** Na Zhang, Jin-lan Long, Yuan Wu, Yong-pin Zhang, Zhi-kun Wu

**Affiliations:** Department of Pharmacy, Guizhou University of Traditional Chinese Medicine, Guiyang, China

**Keywords:** Complete chloroplast genome, phylogenetic analysis, *Indigofera stachyodes*

## Abstract

*Indigofera stachyodes* Lindl. is a traditional medicinal plant in southwestern China. In this study, we report the complete chloroplast genome sequence of *I. stachyodes*, using next-generation sequencing technology. The complete chloroplast genome of *I. stachyodes* was 158,039 bp in length with an overall GC content 35.80%, containing a large single-copy (LSC) region of 88,772 bp, a small single-copy (SSC) region of 18,733 bp, and a pair of inverted repeats (IRs) regions of 25,267 bp. In total, there are 128 genes (83 protein-coding genes (PCGs), eight ribosomal RNA (rRNA) genes, and 37 tRNA genes) in the whole chloroplast genome, including 113 unique genes (78 unique PCGs, 31 unique tRNAs, and four unique rRNAs). The phylogenetic analysis indicated that *I. stachyodes* formed a monophyletic clade with *I. tinctoria* and *I. linifolia*, showing that they have close relationship. The complete chloroplast genome of *I. stachyodes* provides valuable genomic information for the phylogeny, molecular identification and sustainable utilization of this species.

The plant *Indigofera stachyodes* Lindley 1843, which belongs to Papilionoideae of Fabaceae, is a perennial shrub, 1–3 m tall, mainly distributed in Guizhou province, Yunnan province, and Guangxi Zhuang National Autonomous Region of southwestern China, as well as Bhutan, Cambodia, India, Indonesia, Laos, Myanmar, Nepal, Thailand, and Vietnam (Gao and Brian 2010). The roots of *I. stachyodes* are well known as Xue-ren-shen in Chinese, have been traditionally used as a traditional medicine by the Miao people to treat a wide array of human ailments, such as wounds, dysentery, cirrhosis, and rheumatism (Editorial Committee of Chinese Ben-cao 1999). A variety of biological properties including antioxidative, *α*-glucosidase inhibitory, and anti-inflammatory activities have been reported for crude extracts from its roots (Li et al. 2011; Qiu et al. 2013). In this study, we report the first chloroplast genome of *I. stachyodes*, which will provide valuable genomic information for the study of phylogeny, molecular identification and sustainable utilization of this species.

Fresh leaves of *I. stachyodes* were collected from a wild population (106°40′29″ E, 26°26′31″ N) in Huaxi district, Guizhou Province, China. The voucher specimen was deposited in the herbarium of Guizhou University of Traditional Chinese Medicine (Cheng-gang Hu, 2274547063@qq.com) under the voucher number ZN20201026. We isolated the total genomic DNA following a modified CTAB protocol (Doyle 1991). According to the criteria of this protocol, we fragmented the DNA and used an Illumina Hiseq X Ten sequencer to construct the genomic library for Illumina paired-end (PE) sequencing. NOVOplasty v2.7.2 (Dierckxsens et al. 2017) was then used to assemble the complete chloroplast genome of *I. stachyodes*. We also used Geneious v 8.0.2 software to annotate the assembled chloroplast genome (Kearse et al. 2012). The annotated chloroplast genome of *I. stachyodes* was deposited into GenBank with the accession number MZ768851.

The complete chloroplast genome of *I. stachyodes* was 158,039 bp in length with a typical quadripartite structure, and an overall GC content of 35.8%. The assembled genome contained a large single-copy (LSC) region of 88,772 bp, a small single-copy (SSC) region of 18,733 bp, and a pair of inverted repeats (IRs) regions of 25,267 bp. In total, there are 128 genes (83 protein-coding genes (PCGs), eight ribosomal RNA (rRNA) genes, and 37 tRNA genes) in the whole chloroplast genome, including 113 unique genes (78 unique PCGs, 31 unique tRNAs, and four unique rRNAs, respectively).

The maximum-likelihood (ML) phylogenetic tree was constructed based on 17 complete chloroplast genomes (all coding and noncoding sequences) of Papilionoideae species and two complete chloroplast genomes of Caesalpinioideae species as outgroups, using IQ-TREE v1.6.10 (Nguyen et al. 2015) and performed based on a TVM + F+R2 model according to Bayesian’s information criteria using ModelFinder (Kalyaanamoorthy et al. 2017). Ultrafast bootstrap (UFBoot) was used to test branch supports (Hoang et al. 2018) and an SH-like approximate likelihood ratio with 10,000 bootstrap replicates (Figure 1). The phylogenetic analysis indicated that the plants in the same genus, *I. stachyodes*, *I. linifolia*, and *I. tinctoria*, formed a monophyletic clade with 100% bootstrap value, showing that they have a close relationship. Within this monophyletic clade, *I. stachyodes* was most closely related to *I. tinctoria* with 99.3% protein coding sequences identical and 94.9% non-coding sequences identical, compared 98.7% of protein coding sequences and 91.5% of non-coding sequences identical to those of *I. linifolia.* This reported *I. stachyodes* chloroplast genome will provide useful information for molecular identification of close species of *Indigofera*, and also for phylogenetic and evolutionary studies in Fabaceae.

**Figure 1.**
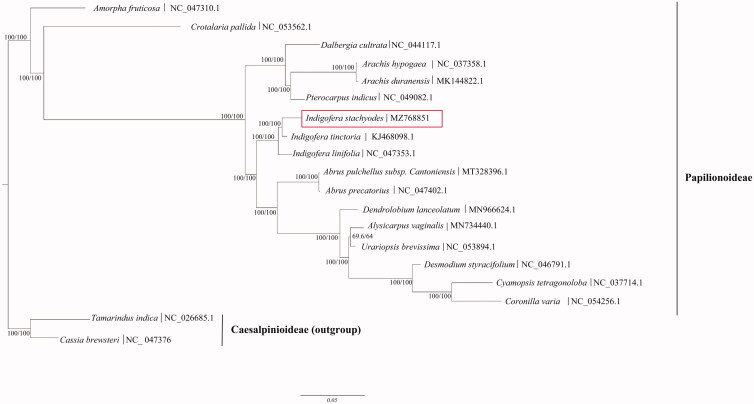
The maximum-likelihood tree based on the complete chloroplast genome sequences of *Indigofera stachyodes* Lindl. and related taxa within the family Fabaceae, branch support values were reported as SH-aLRT/UFBoot. The accession number of GenBank for each species is listed in the figure.

## Data Availability

The genome sequence data that support the findings of this study are openly available in GenBank of NCBI at https://www.ncbi.nlm.nih.gov/ under the accession no MZ768851. The associated BioProject, SRA, and Bio-Sample numbers are PRJNA752685, SRR15367629, and SAMN20606238, respectively.
